# Supplementary material for: Discovery and application of insertion-deletion (INDEL) polymorphisms for QTL mapping of early life-history traits in Atlantic salmon
Source: BMC Genomics. 2010 Mar 8;11:156. doi: 10.1186/1471-2164-11-156 (PMC2838853; doi:10.1186/1471-2164-11-156)
Supplement: Additional file 2 — Information on developed 76 locus single-run INDEL panel in Atlantic salmon. Information on fluorescence labeling, primer concentrations, PCR pooling and links to alignments, INDEL motifs and GENESCAN (Burge and Karlin 1997) predictions of genes/exons are available in html format. [file 1471-2164-11-156-S2.ZIP › Additionalfile2/snpsummary4151.html]

```
Cluster 943 Contig 1

prev  Summary    Contig List  next
```

Size of Consensus sequence = 1796

Number of sequences = 48

Minimum redundancy = 6

Key

A gi|24335353|gb|CA036486.1|CA036486 ssaltc007087 reproductive Salmo salar cDNA, mRNA sequence  
B gi|117510051|gb|EG841810.1|EG841810 EST\_ssal\_eve\_4522 ssaleve thyroid Salmo salar cDNA Salmo salar cDNA clone ssal\_eve\_505\_060\_fwd 3', mRNA sequence  
C gi|15967327|gb|BE518476.2|BE518476 EST00134 Atlantic salmon Lambda Zap Express spleen cDNA library Salmo salar cDNA clone SNN-1056 5' similar to Progesterone receptor binding protein, mRNA sequence  
D gi|84983098|gb|DW533448.1|DW533448 EST\_ssal\_plnb\_2084 plnb Salmo salar cDNA clone ssal\_plnb\_016\_028\_rev 5', mRNA sequence  
E gi|85155864|gb|DW588923.1|DW588923 sbra1-007AA02.g1\_#0023.0 DIAS\_SBRA Salmo salar cDNA 5', mRNA sequence  
F gi|117492327|gb|EG824544.1|EG824544 EST\_ssal\_evd\_26070 ssalevd thymus Salmo salar cDNA Salmo salar cDNA clone ssal\_evd\_533\_366\_fwd 3', mRNA sequence  
G gi|117492325|gb|EG824542.1|EG824542 EST\_ssal\_evd\_26069 ssalevd thymus Salmo salar cDNA Salmo salar cDNA clone ssal\_evd\_533\_366\_rev 5', mRNA sequence  
H gi|117495540|gb|EG827757.1|EG827757 EST\_ssal\_eve\_40273 ssaleve thyroid Salmo salar cDNA Salmo salar cDNA clone ssal\_eve\_554\_307\_fwd 3', mRNA sequence  
I gi|117495541|gb|EG827758.1|EG827758 EST\_ssal\_eve\_40274 ssaleve thyroid Salmo salar cDNA Salmo salar cDNA clone ssal\_eve\_554\_307\_rev 5', mRNA sequence  
J gi|117479308|gb|EG811525.1|EG811525 EST\_ssal\_evd\_31693 ssalevd thymus Salmo salar cDNA Salmo salar cDNA clone ssal\_evd\_542\_017\_fwd 3', mRNA sequence  
K gi|117525112|gb|EG856839.1|EG856839 EST\_ssal\_eve\_50445 ssaleve thyroid Salmo salar cDNA Salmo salar cDNA clone ssal\_eve\_568\_263\_rev 5', mRNA sequence  
L gi|117552852|gb|EG883607.1|EG883607 EST\_ssal\_eve\_26608 ssaleve thyroid Salmo salar cDNA Salmo salar cDNA clone ssal\_eve\_536\_068\_rev 5', mRNA sequence  
M gi|117830498|gb|EG903194.1|EG903194 EST\_ssal\_evf\_41776 ssalevf mixed\_tissue Salmo salar cDNA Salmo salar cDNA clone ssal\_evf\_555\_299\_fwd 3', mRNA sequence  
N gi|117830500|gb|EG903196.1|EG903196 EST\_ssal\_evf\_41777 ssalevf mixed\_tissue Salmo salar cDNA Salmo salar cDNA clone ssal\_evf\_555\_299\_rev 5', mRNA sequence  
O gi|117552853|gb|EG883608.1|EG883608 EST\_ssal\_eve\_26609 ssaleve thyroid Salmo salar cDNA Salmo salar cDNA clone ssal\_eve\_536\_068\_fwd 3', mRNA sequence  
P gi|117469973|gb|EG802192.1|EG802192 EST\_ssal\_evd\_58756 ssalevd thymus Salmo salar cDNA Salmo salar cDNA clone ssal\_evd\_579\_120\_rev 5', mRNA sequence  
Q gi|117472506|gb|EG804725.1|EG804725 EST\_ssal\_evd\_1772 ssalevd thymus Salmo salar cDNA Salmo salar cDNA clone ssal\_evd\_006\_380\_rev 5', mRNA sequence  
R gi|117469974|gb|EG802193.1|EG802193 EST\_ssal\_evd\_58757 ssalevd thymus Salmo salar cDNA Salmo salar cDNA clone ssal\_evd\_579\_120\_fwd 3', mRNA sequence  
S gi|117525113|gb|EG856840.1|EG856840 EST\_ssal\_eve\_50446 ssaleve thyroid Salmo salar cDNA Salmo salar cDNA clone ssal\_eve\_568\_263\_fwd 3', mRNA sequence  
T gi|117524625|gb|EG856352.1|EG856352 EST\_ssal\_eve\_16910 ssaleve thyroid Salmo salar cDNA Salmo salar cDNA clone ssal\_eve\_522\_374\_rev 5', mRNA sequence  
U gi|117530633|gb|EG862087.1|EG862087 EST\_ssal\_eve\_48735 ssaleve thyroid Salmo salar cDNA Salmo salar cDNA clone ssal\_eve\_566\_132\_fwd 3', mRNA sequence  
V gi|117524626|gb|EG856353.1|EG856353 EST\_ssal\_eve\_16911 ssaleve thyroid Salmo salar cDNA Salmo salar cDNA clone ssal\_eve\_522\_374\_fwd 3', mRNA sequence  
W gi|117530622|gb|EG862076.1|EG862076 EST\_ssal\_eve\_48734 ssaleve thyroid Salmo salar cDNA Salmo salar cDNA clone ssal\_eve\_566\_132\_rev 5', mRNA sequence  
X gi|117853294|gb|EG925990.1|EG925990 EST\_ssal\_evf\_28548 ssalevf mixed\_tissue Salmo salar cDNA Salmo salar cDNA clone ssal\_evf\_537\_327\_fwd 3', mRNA sequence  
Y gi|117853295|gb|EG925991.1|EG925991 EST\_ssal\_evf\_28549 ssalevf mixed\_tissue Salmo salar cDNA Salmo salar cDNA clone ssal\_evf\_537\_327\_rev 5', mRNA sequence  
Z gi|117869625|gb|EG942321.1|EG942321 EST\_ssal\_evf\_25658 ssalevf mixed\_tissue Salmo salar cDNA Salmo salar cDNA clone ssal\_evf\_533\_374\_fwd 3', mRNA sequence  
a gi|117449785|gb|EG782004.1|EG782004 EST\_ssal\_evd\_38587 ssalevd thymus Salmo salar cDNA Salmo salar cDNA clone ssal\_evd\_551\_208\_fwd 3', mRNA sequence  
b gi|117840932|gb|EG913628.1|EG913628 EST\_ssal\_evf\_15423 ssalevf mixed\_tissue Salmo salar cDNA Salmo salar cDNA clone ssal\_evf\_519\_056\_fwd 3', mRNA sequence  
c gi|117546551|gb|EG877996.1|EG877996 EST\_ssal\_eve\_21557 ssaleve thyroid Salmo salar cDNA Salmo salar cDNA clone ssal\_eve\_529\_118\_fwd 3', mRNA sequence  
d gi|117833919|gb|EG906615.1|EG906615 EST\_ssal\_evf\_9111 ssalevf mixed\_tissue Salmo salar cDNA Salmo salar cDNA clone ssal\_evf\_510\_206\_fwd 3', mRNA sequence  
e gi|117538340|gb|EG869785.1|EG869785 EST\_ssal\_eve\_30999 ssaleve thyroid Salmo salar cDNA Salmo salar cDNA clone ssal\_eve\_542\_035\_fwd 3', mRNA sequence  
f gi|117822787|gb|EG895483.1|EG895483 EST\_ssal\_evf\_4435 ssalevf mixed\_tissue Salmo salar cDNA Salmo salar cDNA clone ssal\_evf\_504\_093\_fwd 3', mRNA sequence  
g gi|85021411|gb|DW550067.1|DW550067 EST\_ssal\_rgb2\_14486 rgb2 Salmo salar cDNA clone ssal\_rgb2\_524\_041\_rev 5', mRNA sequence  
h gi|117447362|gb|EG779581.1|EG779581 EST\_ssal\_evd\_36406 ssalevd thymus Salmo salar cDNA Salmo salar cDNA clone ssal\_evd\_548\_229\_fwd 3', mRNA sequence  
i gi|117822786|gb|EG895482.1|EG895482 EST\_ssal\_evf\_4434 ssalevf mixed\_tissue Salmo salar cDNA Salmo salar cDNA clone ssal\_evf\_504\_093\_rev 5', mRNA sequence  
j gi|117869626|gb|EG942322.1|EG942322 EST\_ssal\_evf\_25659 ssalevf mixed\_tissue Salmo salar cDNA Salmo salar cDNA clone ssal\_evf\_533\_374\_rev 5', mRNA sequence  
k gi|117840933|gb|EG913629.1|EG913629 EST\_ssal\_evf\_15424 ssalevf mixed\_tissue Salmo salar cDNA Salmo salar cDNA clone ssal\_evf\_519\_056\_rev 5', mRNA sequence  
l gi|117449786|gb|EG782005.1|EG782005 EST\_ssal\_evd\_38588 ssalevd thymus Salmo salar cDNA Salmo salar cDNA clone ssal\_evd\_551\_208\_rev 5', mRNA sequence  
m gi|117546550|gb|EG877995.1|EG877995 EST\_ssal\_eve\_21556 ssaleve thyroid Salmo salar cDNA Salmo salar cDNA clone ssal\_eve\_529\_118\_rev 5', mRNA sequence  
n gi|117447363|gb|EG779582.1|EG779582 EST\_ssal\_evd\_36407 ssalevd thymus Salmo salar cDNA Salmo salar cDNA clone ssal\_evd\_548\_229\_rev 5', mRNA sequence  
o gi|117833918|gb|EG906614.1|EG906614 EST\_ssal\_evf\_9110 ssalevf mixed\_tissue Salmo salar cDNA Salmo salar cDNA clone ssal\_evf\_510\_206\_rev 5', mRNA sequence  
p gi|117510052|gb|EG841811.1|EG841811 EST\_ssal\_eve\_4523 ssaleve thyroid Salmo salar cDNA Salmo salar cDNA clone ssal\_eve\_505\_060\_rev 5', mRNA sequence  
q gi|29316744|gb|CB505518.1|CB505518 ssalmge504218 gut Salmo salar cDNA, mRNA sequence  
r gi|117538342|gb|EG869787.1|EG869787 EST\_ssal\_eve\_31000 ssaleve thyroid Salmo salar cDNA Salmo salar cDNA clone ssal\_eve\_542\_035\_rev 5', mRNA sequence  
s gi|84983099|gb|DW533449.1|DW533449 EST\_ssal\_plnb\_2085 plnb Salmo salar cDNA clone ssal\_plnb\_016\_028\_fwd 3', mRNA sequence  
t gi|29313879|gb|CB502653.1|CB502653 ssalmge503091 gut Salmo salar cDNA, mRNA sequence  
u gi|117492946|gb|EG825163.1|EG825163 EST\_ssal\_evd\_26628 ssalevd thymus Salmo salar cDNA Salmo salar cDNA clone ssal\_evd\_534\_275\_rev 5', mRNA sequence  
v gi|117492947|gb|EG825164.1|EG825164 EST\_ssal\_evd\_26629 ssalevd thymus Salmo salar cDNA Salmo salar cDNA clone ssal\_evd\_534\_275\_fwd 3', mRNA sequence

2 SNPs detected

A B C D E F G H I J K L M N O P Q R S T U V W X Y Z a b c d e f g h i j k l m n o p q r s t u v  cosegregation weighted

521 . . . . - - - - - - G G G G G G G G G G G G G G G G - - - - - G G . G . . . . . . . . . . . . .   2/2 62.50
522 . . . . - - - - - - T T T T T T T T T T T T T T T T - - - - - T T . T . . . . . . . . . . . . .   2/2 62.50
